# Novel Reassortant Highly Pathogenic Avian Influenza (H5N5) Viruses in Domestic Ducks, China

**DOI:** 10.3201/eid1706.101406

**Published:** 2011-06

**Authors:** Min Gu, Wenbo Liu, Yongzhong Cao, Daxin Peng, Xiaobo Wang, Hongquan Wan, Guo Zhao, Quangang Xu, Wei Zhang, Qingqing Song, Yanfang Li, Xiufan Liu

**Affiliations:** Author affiliation: Yangzhou University College of Veterinary Medicine, Yangzhou, People’s Republic of China

**Keywords:** H5N5, highly pathogenic avian influenza, reassortant, domestic ducks, viruses, influenza, China, dispatch

## Abstract

In China, domestic ducks and wild birds often share the same water, in which influenza viruses replicate preferentially. Isolation of 2 novel reassortant highly pathogenic avian influenza (H5N5) viruses from apparently healthy domestic ducks highlights the role of these ducks as reassortment vessels. Such new subtypes of influenza viruses may pose a pandemic threat.

Aquatic birds are considered the natural reservoir for influenza A viruses of all known 16 hemagglutinin (HA) and 9 neuraminidase (NA) subtypes (*1*). Influenza viruses replicate preferentially in the cells lining the intestinal tracts of wild waterfowl, usually causing no clinical signs. Excretion of substantial amounts of virus in the feces can infect wild and domestic birds by waterborne transmission (*1*). In the People’s Republic of China, domestic ducks raised in the traditional free-range system often share water with wild aquatic birds. Moreover, domestic ducks are often in close contact with poultry, livestock, and humans in the same village or farm. Therefore, domestic ducks play a major role in the ecology of influenza viruses (*2*) and can act as potential vessels for genetic reassortment (*3*). Systematic surveillance of influenza viruses in domestic ducks could provide timely and valuable epidemiologic information and should be continued.

## The Study

As part of routine surveillance for avian influenza viruses from December 2008 through January 2009 in eastern China, tracheal and cloacal swab samples from apparently healthy domestic ducks in live poultry markets were collected for virus isolation and identification as described (*4*). From these samples, 2 influenza (H5N5) viruses—A/duck/eastern China/008/2008 (008 [H5N5]) and A/duck/eastern China/031/2009 (031 [H5N5])—were detected in mallard ducks (*Anas platyrhynchos*).

These 2 viruses grew efficiently in eggs and in MDCK cells, each with virus titers >8 log_10_ 50% egg infectious dose (EID_50_)/mL or 8 log_10_ 50% tissue culture infectious dose/mL (Table 1). The intravenous pathogenicity index for chickens and 50% lethal dose for mice were 2.6 and 10^4.0^ EID_50_ for 008 (H5N5), 2.5 and 10^5.4^ EID_50_ for 031 (H5N5), respectively (Table 1). Therefore, both novel influenza subtype H5N5 viruses were assumed to be highly pathogenic for chickens and moderately virulent for mice (*5*).

**Table 1:** Characteristics of 2 novel avian influenza (H5N5) viruses isolated from domestic ducks, China, December 2008–January 2009*

Virus	Characteristics					Virus replication in experimentally infected mice, no. virus-positive mice/no. tested mice (mean titer + SD)†					
	IVPI	EID_50_	TCID_50_	MLD_50_‡		Tissue	dpi 2	dpi 4	dpi 6	dpi 8	dpi 10
008	2.6	10^8.5^	10^8.6^	10^4.0^		Lung	1/2 (3.2 ± 0)	2/2 (3.8 ± 0.4)	2/2 (4.7 ± 0.4)	2/2 (4.4 ± 0.5)	0/2
						Brain	0/2	0/2	0/2	2/2 (3.8 ± 1.2)	1/2 (3.4 ± 0)
						Heart	1/2 (2.0 ± 0)	2/2 (2.6 ± 0.5)	2/2 (3.1 ± 0.1)	0/2	0/2
						Spleen	0/2	0/2	0/2	0/2	1/2 (2.3 ± 0)
						Liver	0/2	0/2	1/2 (2.2 ± 0)	0/2	0/2
						Kidney	0/2	0/2	0/2	0/2	0/2
031	2.5	10^8.3^	10^8.5^	10^5.4^		Lung	1/2 (3.0 ± 0)	1/2 (3.3 ± 0)	2/2 (4.1 ± 0.2)	2/2 (4.5 ± 0.3)	1/2 (2.6 ± 0)
						Brain	0/2	0/2	0/2	1/2 (3.4 ± 0)	0/2
						Heart	0/2	1/2 (2.5 ± 0)	1/2 (3.0 ± 0)	0/2	0/2
						Spleen	0/2	0/2	0/2	0/2	0/2
						Liver	0/2	0/2	0/2	0/2	0/2
						Kidney	0/2	0/2	1/2 (2.1 ± 0)	0/2	0/2

*EID_50_, 50% egg infectious dose; IVPI, intravenous pathogenicity index (determined in chickens); TCID_50_, 50% tissue culture infectious dose (determined in MDCK cells); MLD_50_, 50% lethal dose in mice (expressed as the EID_50_ value corresponding to 1 LD_50_); dpi, day postinoculation; 008, A/duck/eastern China/008/2008; 031, A/duck/eastern China/031/2009. †Mice were inoculated with 10^3.5^ EID_50_; 2 infected mice were euthanized every other day for virus detection. Virus titers were expressed as log_10_ EID_50_/g. ‡Influenza A viruses with MLD_50_ <10^_3.0_^ EID_50_ were considered of high pathogenicity, MLD_50_ >10^6.5^ EID_50_ were considered of low pathogenicity (*4*).

When mice were inoculated with a sublethal dose of 10^3.5^ EID_50_, each influenza subtype H5N5 virus was able to replicate without prior adaptation. The highest virus titers were detected in the mouse lung. The viruses were able to spread to the brain and heart. Furthermore, influenza virus 008 (H5N5) was isolated from the spleen and liver, and influenza virus 031 (H5N5) was detected in the kidney (Table 1). Microscopic findings in infected mice were interstitial pneumonia with various amounts of erythrocytes in alveolar lumens, hyperemia and lymphocyte infiltration of meningeal veins and cardiac muscles, low numbers of lymphocytes in periarterial lymphatic sheaths and macrophage recruitment in the spleen, lymphocyte infiltration in the liver, and slight congestion in the renal cortex and glomerulus (data not shown).

Genomic analysis showed that the influenza viruses 008 (H5N5) and 031 (H5N5) were highly homologous with each other, sharing 99.2%–99.7% nt identities among the 8 gene segments except for the polymerase acidic protein (PA) gene (94.1%). Another 3 viruses isolated from the same surveillance study— A/duck/eastern China/108/2008 of H5N1 subtype (108 [H5N1]), A/duck/eastern China/909/2009 of H5N1 subtype (909 [H5N1]), and A/duck/Yangzhou/013/2008 of H6N5 subtype (013 [H6N5])—were more closely related to the novel influenza subtype H5N5 viruses than were those in GenBank (Table 2).

**Table 2:** Influenza viruses with highest nucleotide identity to each gene of 008 and 031*

Gene segment		Closest viruses in GenBank		Closest viruses isolated during surveillance, China, December 2008–January 2009	
		Strain	Nucleotide identity, %	Strain	Nucleotide identity, %
PB2	008	A/duck/Guangxi/xa/2001 (H5N1)	96.0	108	100
	031		95.9	909	99.8
PB1	008	A/duck/Hokkaido/Vac-1/04 (H5N1)	97.2	031	99.5
	031		97.1	909	99.7
PA	008	A/chicken/Hunan/8/2008 (H5N1)	98.1	013	99.7
	031	A/wild duck/Hunan/021/2005 (H5N1)	98.0	108	99.5
HA	008	A/wild duck/Hunan/211/2005 (H5N1)	97.7	108	99.9
	031		97.8	008 and108	99.4
NP	008	A/chicken/Jilin/hk/2004 (H5N1)	96.1	108	99.8
	031		96.5	108	99.6
NA	008	A/mallard/Switzerland/WV4060167/2006 (H3N5)	95.8	031	99.2
	031			008	99.2
M	008	A/China/GD01/2006 (H5N1)	99.1	031 and 909	99.8
	031			909	100
NS	008	A/wild duck/Hunan/021/2005 (H5N1)	97.9	108	99.5
	031		98.2	909	99.9

*PB, polybasic protein; 008, A/duck/eastern China/008/2008(H5N5); 108, A/duck/eastern China/108/2008(H5N1); 031, A/duck/eastern China/031/2009(H5N5); 909, A/duck/eastern China/909/2009(H5N1); 013, A/duck/Yangzhou/013/2008(H6N5); PA, polymerase acidic protein; HA, hemagglutinin; NP, nucelocapsid protein; NA, neuraminidase; M, matrix protein; NS, nonstructural protein.

**Figure 1:**
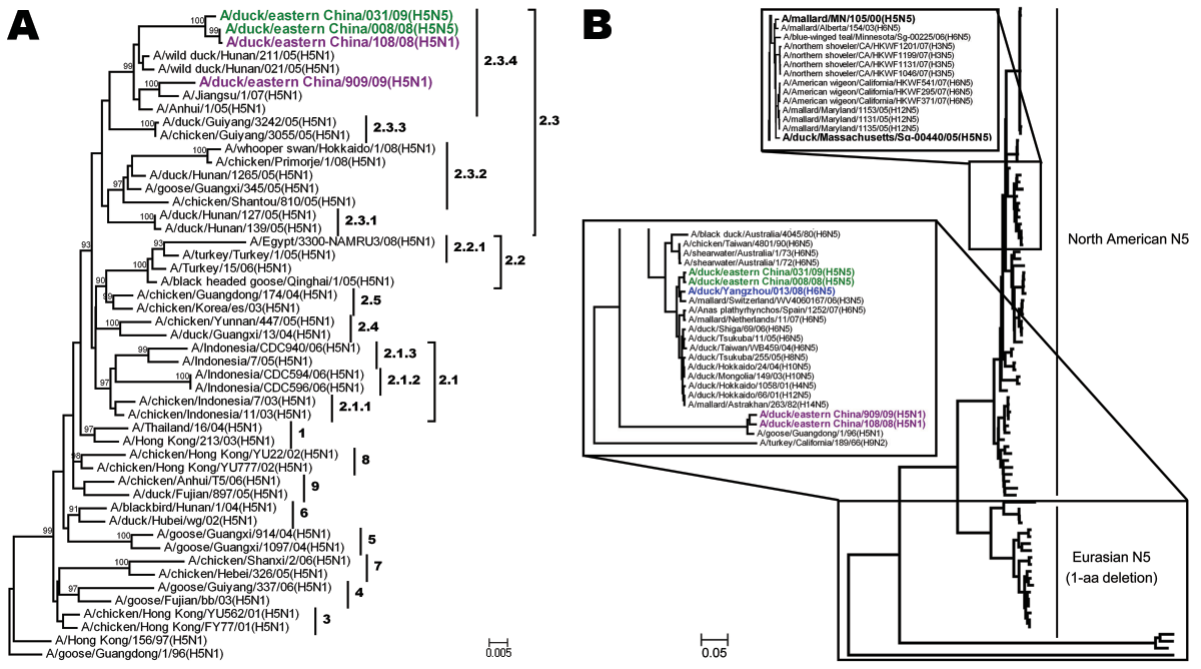
Phylogenetic trees of hemagglutinin (A) and neuraminidase (B) genes of novel avian influenza (H5N5) viruses isolated from domestic ducks in the People’s Republic of China, December 2008–January 2009, with reference sequences. Green, A/duck/eastern China/008/2008 (H5N5) and A/duck/eastern China/031/2009 (H5N5); purple, A/duck/eastern China/108/2008 (H5N1) and A/duck/eastern China/909/2009 (H5N1); blue, A/duck/Yangzhou/013/2008 (H6N5); **boldface**, other H5N5 influenza viruses available from GenBank. Inset boxes in panel B indicate correspondence between thumbnail and panorama of related perspectives. Trees were generated by applying the neighbor-joining method in MEGA 4.0 (www.megasoftware.net) on the basis of full-length coding sequences. Numbers above or below the branch nodes indicate bootstrap values. Scale bars indicate branch length based on number of nucleotide substitutions per site.

As outlined by the World Health Organization/World Organisation for Animal Health/Food and Agriculture Organization unified nomenclature system for subtype H5N1 highly pathogenic avian influenza (HPAI) viruses (*6*), the HA genes of the 2 subtype H5N5 viruses were classified into clade 2.3.4 (Figure 1, panel A), which has been the prevalent lineage in southern China since 2005 (*7**–**9*). In addition to the typical residues Q226 and G228 in HA, which confer receptor preference for SAα2,3Gal, influenza viruses 008 (H5N5) and 031 (H5N5) simultaneously carried an S227R mutation in the receptor-binding pocket. A recent report (*10*) indicated that the S227N substitution accompanied with deglycosylation at residue 158 could substantially increase the affinity of HA for SAα2,6Gal without reducing its binding affinity for SAα2,3Gal. Whether this S227R variation with the changed residual polarity affects the receptor-binding property deserves further investigation.

To construct the NA tree, we retrieved 90 complete N5 sequences from GenBank, including the only 2 influenza subtype H5N5 viruses from the United States: A/mallard/MN/105/2000 and A/duck/Massachusetts/sg-00440/2005. The N5 viruses were grouped into 2 lineages—North American and Eurasian—in accordance with their geographic distribution (Figure 1, panel B). The 2 subtype H5N5 isolates from China belonged to the Eurasian lineage, whereas the 2 from the United States clustered within the North American lineage. In addition, 1 aa deletion at residue 42, located in the stalk region of NA, was identified in all Eurasian, but not in the North American, strains.

**Figure 2:**
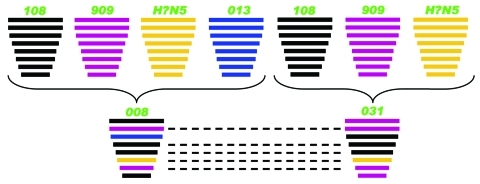
Putative genomic compositions of the novel avian influenza (H5N5) viruses isolated from domestic ducks in the People’s Republic of China, December 2008–January 2009, with their possible donors. The 8 gene segments (from top to bottom) in each virus are polymerase basic protein 2, polymerase basic protein 1, polymerase acidic protein, hemagglutinin (HA), nucleocapsid protein, neuraminidase, matrix protein, and nonstructural protein. Each color indicates a separate virus background. Dashed lines indicate high sequence identity and suggest a second possibility that the 2 influenza (H5N5) viruses could be donors of some gene segments for each other. 108, A/duck/eastern China/108/2008 (H5N1); 909, A/duck/eastern China/909/2009 (H5N1); 008, A/duck/eastern China/008/2008 (H5N5); 031, A/duck/eastern China/031/2009 (H5N5); 013, A/duck/Yangzhou/013/2008 (H6N5). H?N5 denotes possible parental viruses of unidentified HA subtype but N5 subtype. The simplified schematic illustration is based on nucleotide-distance comparison and phylogenetic analysis.

Although the PA genes of influenza viruses 008 (H5N5) and 031 (H5N5) diverged to assemble respectively with influenza viruses 013 (H6N5) and 108 (H5N1), the novel influenza subtype H5N5 viruses aggregated closely with recent Eurasian subtype H5N1 viruses, especially influenza viruses 108 (H5N1) and 909 (H5N1) in the trees of HA (Figure 1, panel A), polymerase basic protein (PB) 2, PB1, nucleocapsid protein, matrix protein, and nonstructural protein genes (Technical Appendix). For NA, spatiotemporal correlation indicates that influenza virus 013 (H6N5), rather than its phylogenetically equidistant counterpart A/mallard/Switzerland/WV4060167/2006(H3N5), might be the N5 donor (Figure 1, panel B). However, because of the relatively low (95%) sequence similarity, it is also possible that Eurasian viruses of an unidentified HA subtype (H?N5), not detected in our epidemiologic survey, could provide the NA genes. Therefore, we speculate that influenza viruses 008 (H5N5) and 031 (H5N5) may be reassortants between contemporary Eurasian subtype H5N1 and some subtype H?N5 and/or H6N5 avian influenza viruses with distant evolutionary relationship with the 2 subtype H5N5 viruses from the United States (Figure 1; Technical Appendix). In addition, regarding the especially high sequence identities of PB1, HA, nucleocapside protein, NA, and matrix protein genes exclusively between influenza viruses 008 (H5N5) and 031 (H5N5), the possibility that the 2 subtype H5N5 viruses donated some gene segments to each other cannot be excluded (Figure 2).

## Conclusions

The 2 novel HPAI (H5N5) viruses isolated and characterized in this study are most likely reassortants of recent Eurasian viruses sharing approximate spatiotemporal distribution. It is less likely that they were introduced through intercontinental transmission of subtype H5N5 strains from North America. Considering the endemicity that clade 2.3.4 subtype H5N1 viruses have gained in China since 2005 (*7**–**9*), it is plausible that subtype H5N1 viruses have provided the backbone for generating the novel subtype H5N5 viruses instead of the opposite gene flow.

Ducks have been considered “Trojan horses” for influenza (H5N1) because of their pivotal role in virus propagation and evolution (*11**–**13*). In our study, the 2 reassortant influenza viruses (008 [H5N5] and 031 [H5N5]) and their 3 possible parent viruses (108 [H5N1], 909 [H5N1], and 013 [H6N5]) were all isolated from apparently healthy domestic ducks. We speculate that domestic ducks may serve as reassortant vessels for creating new subtypes of influenza viruses. In view of the practice of raising ducks in a free-range system, these novel strains could be transmitted to other domestic poultry and even humans. There is evidence that these subtype H5N5 viruses have been transmitted to terrestrial poultry (Zhao et al., unpub. data). Thus, the role of domestic ducks in the influenza virus ecosystem should not be neglected. Systematic surveillance should be instituted to identify emerging HPAI (H5N5) viruses and to reduce their potential threat to animal and human health.

## Supplementary Material

Technical Appendix Phylogenetic trees and reference sequences.

## References

[1] Webster RG, Bean WJ, Gorman OT, Chambers TM, Kawaoka Y. Evolution and ecology of influenza A viruses. Microbiol Rev. 1992;56:152–79. 1579108 10.1128/mr.56.1.152-179.1992 PMC372859

[2] Huang K, Bahl J, Fan XH, Vijaykrishna D, Cheung CL, Webby RJ, Establishment of an H6N2 influenza virus lineage in domestic ducks in southern China. J Virol. 2010;84:6978–86. 10.1128/JVI.00256-10 20463062 PMC2898240

[3] Kim HR, Park CK, Oem JK, Bae YC, Choi JG, Lee OS, Characterization of H5N2 influenza viruses isolated in South Korea and their influence on the emergence of a novel H9N2 influenza virus. J Gen Virol. 2010;91:1978–83. 10.1099/vir.0.021238-0 20392898

[4] Zhang P, Tang Y, Liu X, Peng D, Liu W, Liu H, Characterization of H9N2 influenza viruses isolated from vaccinated flocks in an integrated broiler chicken operation in eastern China during a 5-year period (1998–2002). J Gen Virol. 2008;89:3102–12. 10.1099/vir.0.2008/005652-0 19008399

[5] Katz JM, Lu X, Tumpey TM, Smith CB, Shaw MW, Subbarao K. Molecular correlates of influenza A H5N1 virus pathogenesis in mice. J Virol. 2000;74:10807–10. 10.1128/JVI.74.22.10807-10810.2000 11044127 PMC110957

[6] WHO/OIE/FAO H5N1 Evolution Working Group. Toward a unified nomenclature system for highly pathogenic avian influenza virus (H5N1). Emerg Infect Dis. 2008;14:e1. 18598616 10.3201/eid1407.071681 PMC2600337

[7] Chen H, Smith GJ, Zhang SY, Qin K, Wang J, Li KS, Avian flu: H5N1 virus outbreak in migratory waterfowl. Nature. 2005;436:191–2. 10.1038/nature03974 16007072

[8] Smith GJ, Fan XH, Wang J, Li KS, Qin K, Zhang JX, Emergence and predominance of an H5N1 influenza variant in China. Proc Natl Acad Sci U S A. 2006;103:16936–41. 10.1073/pnas.0608157103 17075062 PMC1636557

[9] Smith GJ, Vijaykrishna D, Ellis TM, Dyrting KC, Leung YH, Bahl J, Characterization of avian influenza viruses A (H5N1) from wild birds, Hong Kong, 2004–2008. Emerg Infect Dis. 2009;15:402–7. 10.3201/eid1503.081190 19239752 PMC2666293

[10] Yen HL, Aldridge JR, Boon AC, Ilyushina NA, Salomon R, Hulse-Post DJ, Changes in H5N1 influenza virus hemagglutinin receptor binding domain affect systemic spread. Proc Natl Acad Sci U S A. 2009;106:286–91. 10.1073/pnas.0811052106 19116267 PMC2629220

[11] Hulse-Post DJ, Sturm-Ramirez KM, Humberd J, Seiler P, Govorkova EA, Krauss S, Role of domestic ducks in the propagation and biological evolution of highly pathogenic H5N1 influenza viruses in Asia. Proc Natl Acad Sci U S A. 2005;102:10682–7. 10.1073/pnas.0504662102 16030144 PMC1180796

[12] Kim JK, Negovetich NJ, Forrest HL, Webster RG. Ducks: the “Trojan horses” of H5N1 influenza. Influenza Other Respir Viruses. 2009;3:121–8. 10.1111/j.1750-2659.2009.00084.x 19627369 PMC2749972

[13] Takakuwa H, Yamashiro T, Le MQ, Phuong LS, Ozaki H, Tsunekuni R, Possible circulation of H5N1 avian influenza viruses in healthy ducks on farms in northern Vietnam. Microbiol Immunol. 2010;54:58–62. 10.1111/j.1348-0421.2009.00170.x 20055944

