# Supplementary material for: Novel Reassortant Highly Pathogenic Avian Influenza (H5N5) Viruses in Domestic Ducks, China
Source: Emerg Infect Dis. 2011 Jun;17(6):1060–3. doi: 10.3201/eid1706.101406 (PMC3358203; doi:10.3201/eid1706.101406)
Supplement: Technical Appendix — Phylogenetic trees and reference sequences. [file 10-1406-Techapp_4p.pdf]

Article DOI: 10.3201/eid1706.101406

Novel Reassortant Highly Pathogenic Avian Influenza (H5N5) Viruses in Domestic Ducks, China

Technical Appendix

Shown on the following pages are phylogenetic trees of the polymerase basic protein (PB) 2, PB1, polymerase acidic protein (PA), nucleocapsid protein (NP), matrix protein (M), and nonstructural protein (NS) genes of the novel avian influenza (H5N5) viruses isolated from domestic ducks in the People's Republic of China, December 2008–January 2009, with reference sequences. Green, 2 subtype H5N5 viruses (A/duck/eastern China/008/2008 and A/duck/eastern China/031/2009); purple, 2 subtype H5N1 viruses (A/duck/eastern China/108/2008 and A/duck/eastern China/909/2009); blue, 1 subtype H6N5 virus (A/duck/Yangzhou/013/2008); **boldface,** other subtype H5N5 viruses available from GenBank. Trees were generated by applying the neighbor-joining method in MEGA 4.0 (www.megasoftware.net) on the basis of full-length coding sequences. Numbers above or below the branch nodes indicate bootstrap values. Scale bars indicate branch length based on the number of nucleotide substitutions per site.

PB2

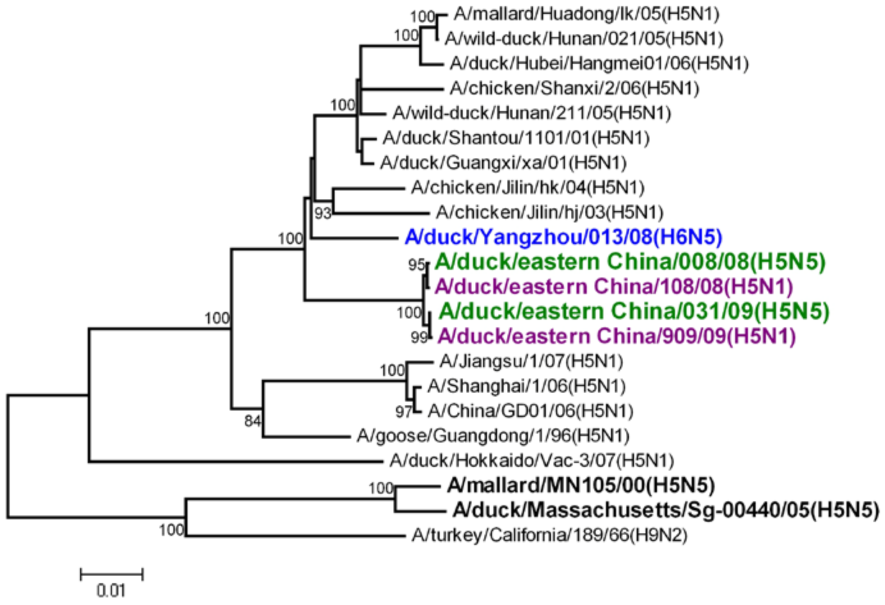
A/mallard/Huadong/lk/05(H5N1)
A/wild-duck/Hunan/021/05(H5N1)
A/duck/Hubei/Hangmei01/06(H5N1)
A/chicken/Shanxi/2/06(H5N1)
A/wild-duck/Hunan/211/05(H5N1)
A/duck/Shantou/1101/01(H5N1)
A/duck/Guangxi/xa/01(H5N1)
A/chicken/Jilin/hk/04(H5N1)
A/chicken/Jilin/hj/03(H5N1)
A/duck/Yangzhou/013/08(H6N5)
A/duck/eastern China/008/08(H5N5)
A/duck/eastern China/108/08(H5N1)
A/duck/eastern China/031/09(H5N5)
A/duck/eastern China/909/09(H5N1)
A/Jiangsu/1/07(H5N1)
A/Shanghai/1/06(H5N1)
A/China/GD01/06(H5N1)
A/goose/Guangdong/1/96(H5N1)
A/duck/Hokkaido/Vac-3/07(H5N1)
A/mallard/MN105/00(H5N5)
A/duck/Massachusetts/Sg-00440/05(H5N5)
A/turkey/California/189/66(H9N2)
0.01

PB1

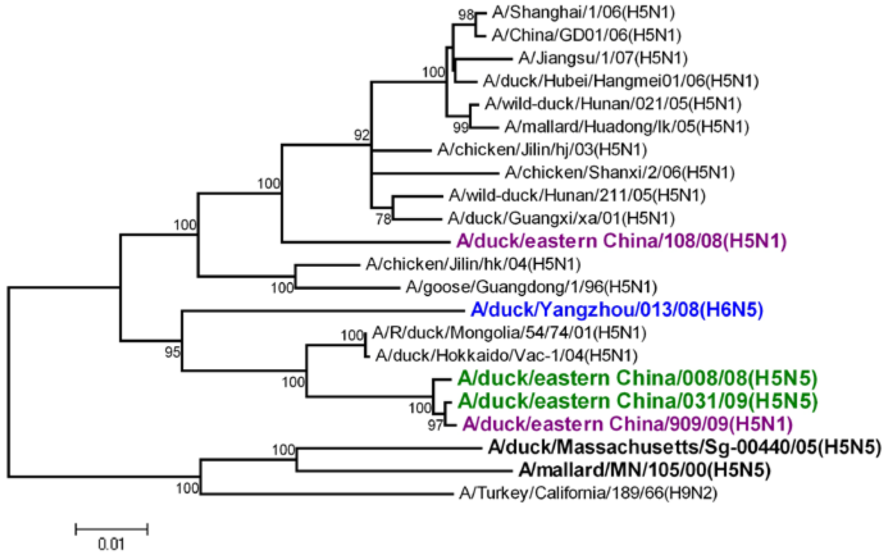
A/Shanghai/1/06(H5N1)
A/China/GD01/06(H5N1)
A/Jiangsu/1/07(H5N1)
A/duck/Hubei/Hangmei01/06(H5N1)
A/wild-duck/Hunan/021/05(H5N1)
A/mallard/Huadong/lk/05(H5N1)
A/chicken/Jilin/hj/03(H5N1)
A/chicken/Shanxi/2/06(H5N1)
A/wild-duck/Hunan/211/05(H5N1)
A/duck/Guangxi/xa/01(H5N1)
A/duck/eastern China/108/08(H5N1)
A/chicken/Jilin/hk/04(H5N1)
A/goose/Guangdong/1/96(H5N1)
A/duck/Yangzhou/013/08(H6N5)
A/R/duck/Mongolia/54/74/01(H5N1)
A/duck/Hokkaido/Vac-1/04(H5N1)
A/duck/eastern China/008/08(H5N5)
A/duck/eastern China/031/09(H5N5)
A/duck/eastern China/909/09(H5N1)
A/duck/Massachusetts/Sg-00440/05(H5N5)
A/mallard/MN/105/00(H5N5)
A/Turkey/California/189/66(H9N2)
0.01

PA

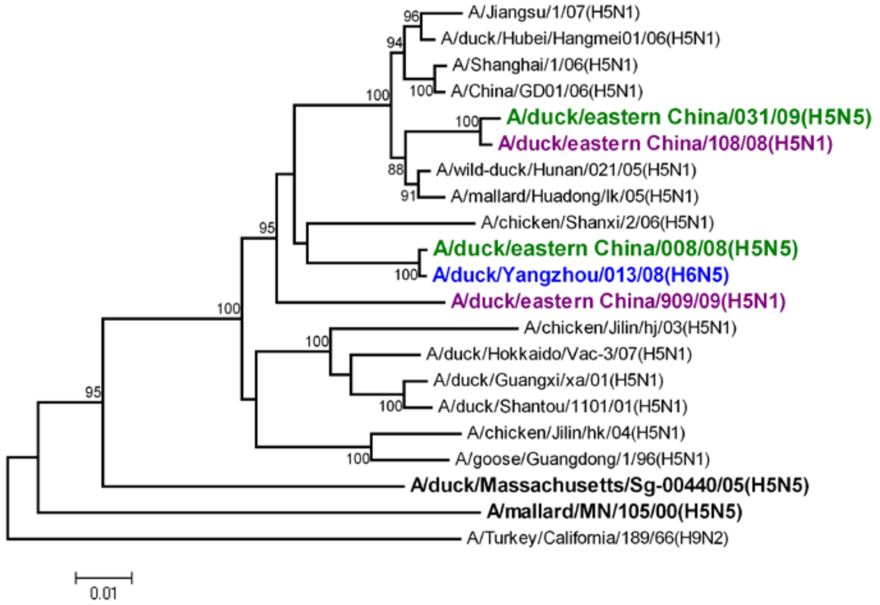
A/Jiangsu/1/07(H5N1)
A/duck/Hubei/Hangmei01/06(H5N1)
A/Shanghai/1/06(H5N1)
A/China/GD01/06(H5N1)
A/duck/eastern China/031/09(H5N5)
A/duck/eastern China/108/08(H5N1)
A/wild-duck/Hunan/021/05(H5N1)
A/mallard/Huadong/lk/05(H5N1)
A/chicken/Shanxi/2/06(H5N1)
A/duck/eastern China/008/08(H5N5)
A/duck/Yangzhou/013/08(H6N5)
A/duck/eastern China/909/09(H5N1)
A/chicken/Jilin/hj/03(H5N1)
A/duck/Hokkaido/Vac-3/07(H5N1)
A/duck/Guangxi/xa/01(H5N1)
A/duck/Shantou/1101/01(H5N1)
A/chicken/Jilin/hk/04(H5N1)
A/goose/Guangdong/1/96(H5N1)
A/duck/Massachusetts/Sg-00440/05(H5N5)
A/mallard/MN/105/00(H5N5)
A/Turkey/California/189/66(H9N2)
0.01

NP

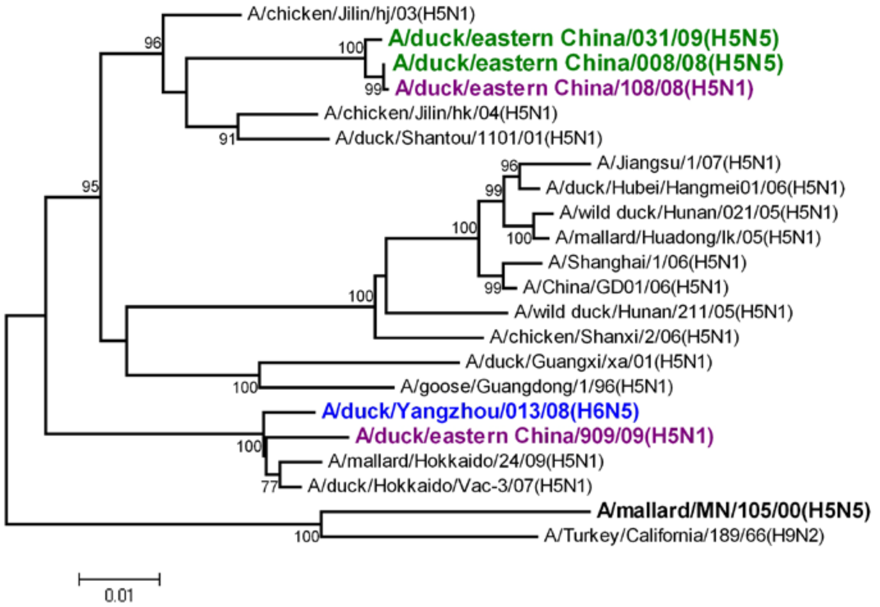
A/chicken/Jilin/hj/03(H5N1)
A/duck/eastern China/031/09(H5N5)
A/duck/eastern China/008/08(H5N5)
A/duck/eastern China/108/08(H5N1)
A/chicken/Jilin/hk/04(H5N1)
A/duck/Shantou/1101/01(H5N1)
A/Jiangsu/1/07(H5N1)
A/duck/Hubei/Hangmei01/06(H5N1)
A/wild duck/Hunan/021/05(H5N1)
A/mallard/Huadong/lk/05(H5N1)
A/Shanghai/1/06(H5N1)
A/China/GD01/06(H5N1)
A/wild duck/Hunan/211/05(H5N1)
A/chicken/Shanxi/2/06(H5N1)
A/duck/Guangxi/xa/01(H5N1)
A/goose/Guangdong/1/96(H5N1)
A/duck/Yangzhou/013/08(H6N5)
A/duck/eastern China/909/09(H5N1)
A/mallard/Hokkaido/24/09(H5N1)
A/duck/Hokkaido/Vac-3/07(H5N1)
A/mallard/MN/105/00(H5N5)
A/Turkey/California/189/66(H9N2)
0.01

M

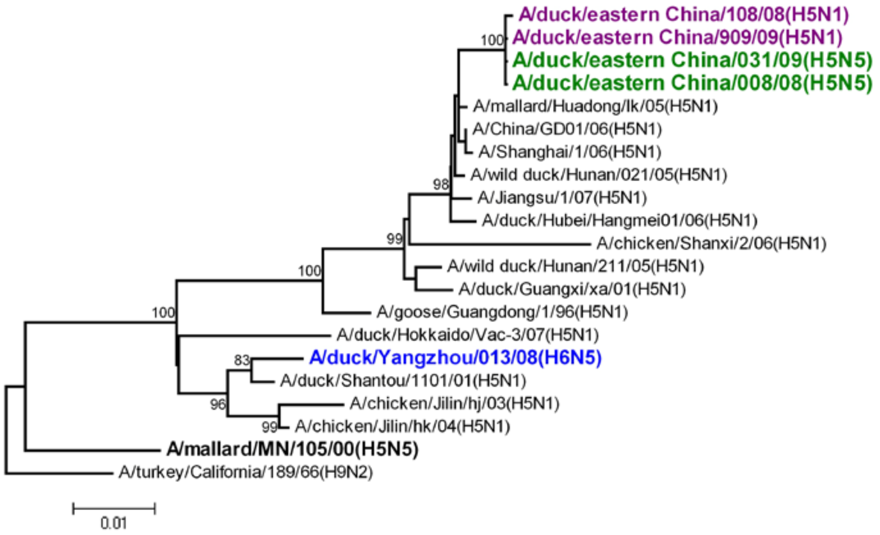
A/duck/eastern China/108/08(H5N1)
A/duck/eastern China/909/09(H5N1)
A/duck/eastern China/031/09(H5N5)
A/duck/eastern China/008/08(H5N5)
A/mallard/Huadong/lk/05(H5N1)
A/China/GD01/06(H5N1)
A/Shanghai/1/06(H5N1)
A/wild duck/Hunan/021/05(H5N1)
A/Jiangsu/1/07(H5N1)
A/duck/Hubei/Hangmei01/06(H5N1)
A/chicken/Shanxi/2/06(H5N1)
A/wild duck/Hunan/211/05(H5N1)
A/duck/Guangxi/xa/01(H5N1)
A/goose/Guangdong/1/96(H5N1)
A/duck/Hokkaido/Vac-3/07(H5N1)
A/duck/Yangzhou/013/08(H6N5)
A/duck/Shantou/1101/01(H5N1)
A/chicken/Jilin/hj/03(H5N1)
A/chicken/Jilin/hk/04(H5N1)
A/mallard/MN/105/00(H5N5)
A/turkey/California/189/66(H9N2)
0.01

NS

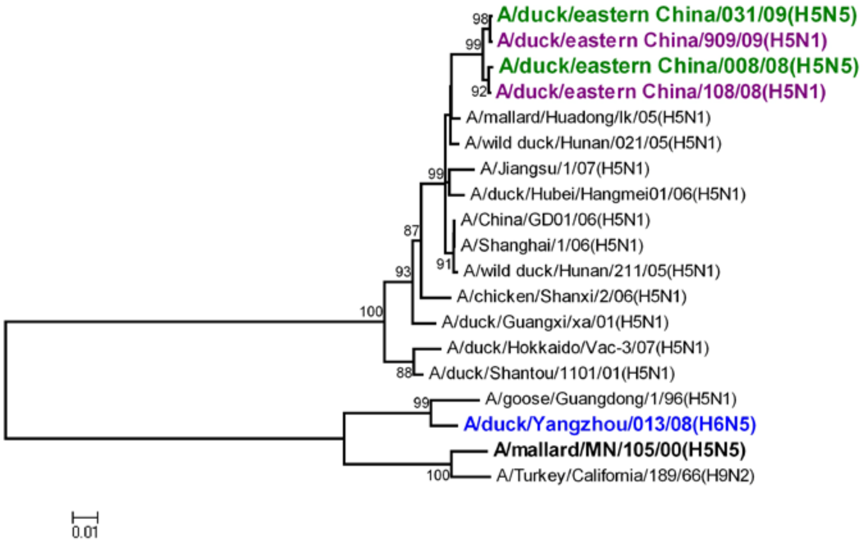
A/duck/eastern China/031/09(H5N5)
A/duck/eastern China/909/09(H5N1)
A/duck/eastern China/008/08(H5N5)
A/duck/eastern China/108/08(H5N1)
A/mallard/Huadong/lk/05(H5N1)
A/wild duck/Hunan/021/05(H5N1)
A/Jiangsu/1/07(H5N1)
A/duck/Hubei/Hangmei01/06(H5N1)
A/China/GD01/06(H5N1)
A/Shanghai/1/06(H5N1)
A/wild duck/Hunan/211/05(H5N1)
A/chicken/Shanxi/2/06(H5N1)
A/duck/Guangxi/xa/01(H5N1)
A/duck/Hokkaido/Vac-3/07(H5N1)
A/duck/Shantou/1101/01(H5N1)
A/goose/Guangdong/1/96(H5N1)
A/duck/Yangzhou/013/08(H6N5)
A/mallard/MN/105/00(H5N5)
A/Turkey/California/189/66(H9N2)
0.01
